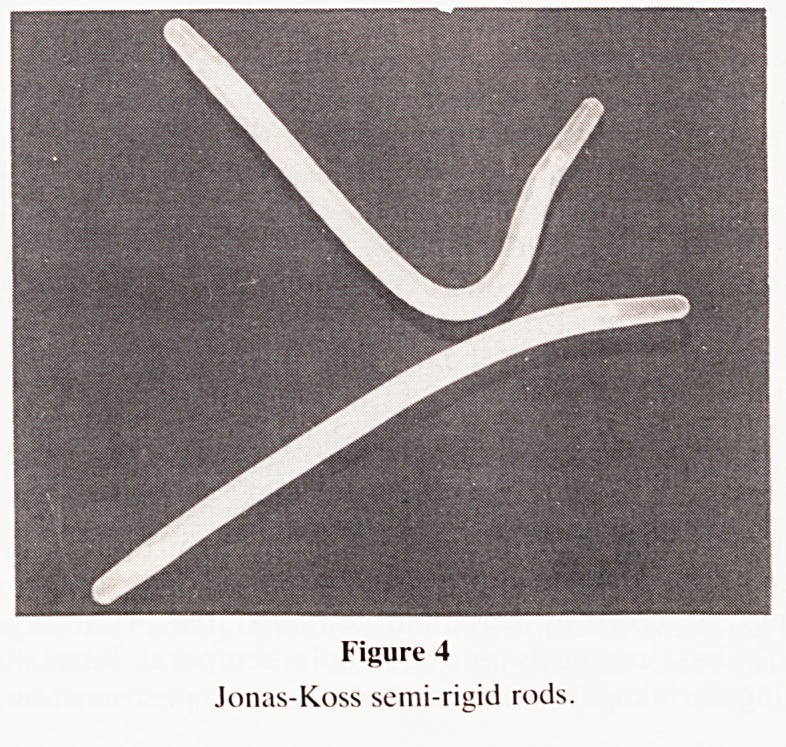# Penile Rods, the Southmead Experience

**Published:** 1990-09

**Authors:** S. Gepi-Attee, J. C. Gingell

**Affiliations:** Southmead Hospital, Bristol; Southmead Hospital, Bristol


### Penile Rods – The Southmead experience

**S. Gepi-Attee FRCSI and J. C. Gingell FRCS, FRCS (Ed)**
Southmead Hospital, Bristol

### INTRODUCTION

This is a review of 34 patients who had penile rod insertion for erectile dysfunction between May 1980 and February 1988 in the Urological service of Southmead Hospital. During this period significant advances have occurred in our investigation and management of impotence. In particular the discovery of intracorporeal papaverine. This might have been considered to reduce the indication for prostheses but on the other hand the successful institution of such a clinic at Southmead has attracted greater numbers of patients with erectile failure to this hospital and the impotence clinic now has an annual attendance of greater than 300 patients. The annual numbers of prostheses inserted over this period has increased correspondingly.

### METHODS

Clinical notes of all patients who had been operated on during this period were recovered and studied. The indications for treatment, the underlying cause, associated medical conditions and drug history were noted. Investigations which were undertaken to confirm the category of impotence were also noted. The detail of the surgical procedure including type of incision, the type of prosthesis, the immediate post-operative result and complications were assessed. Finally the overall patient-assessment of the result was obtained by filling a detailed questionnaire. The patients were invited to the out-patients department for an interview based on the questionnaire or they filled the questionnaire sent to them by post. The results of this survey are presented here.

### RESULT

Thirty-four patients were operated on during this period. The mean age was 51.5 years (S.D. 10.9) with a range of 28 to 74 years (figure 1). On average we operated on 4 patients annually. The indication for the operation was impotence i.e. failure of adequate erection for sexual intercourse. The underlying causes for impotence were varied. Twenty-three (67.6%) were diabetics (figure 2) of which 15 were insulin dependent with mean age 46 (S.D. 10) and mean duration of diabetes 18 years (S.D. 10). Duration of impotence in this group averaged 3 years. There were 8 diabetics of maturity onset with a higher average age 58 years (S.D. 8) and far shorter duration of diabetes (6 years S.D. 4) though with longer duration of impotence (5 years). Six diabetics had co-existing associated Peyronie's plaques. Of the non-diabetic group 4 followed surgical operations, 3 had severe associated medical conditions whilst 2 had underlying Peyronie's plaques only. One was post-priapism and 1 was probably of psychogenic origin. The average duration of hospitalization was 7.15 days (S.D. 2.62). The surgical approach was predominantly peno-scrotal (figure 3) though a coronal incision was used in a minority of cases (n = 9). Nearly all the prostheses were semi-rigid silver wire core silicone prostheses of the Jonas-Koss type (figure 4). One Subrini type and one inflatable Brantley-Scott type were also used. The procedure was always done under general anaesthesia with penile block for post-operative analgesia. Intravenous antibiotics were given with induction and continued for 48 hours thereafter. This was followed by one week's course of oral antibiotics usually trimethoprim. The patient was advised against having intercourse before out-patient review in about 3 to 4 weeks. Subsequently they were seen at about 6 months to assess tolerance and function. Complications were few. Two patients had their rods removed subsequently – one at another hospital because of sepsis and the other for lack of acceptance by his partner despite a satisfactory surgical result. There were 2 minor superficial wound infections and one haematoma all of which were treated conservatively. Twenty-six of the 34 (76%) co-operated with the questionnaire evaluation. Of these 24 were having intercourse regularly the remaining 2 less frequently – one because of an ill partner. Significant pain was experienced by 17 patients during the immediate post-operative period of average duration 3 weeks and of varying intensity. Once the pain settled there was no discomfort during intercourse. Three patients complained of some difficulty regarding concealment and hence embarrassment. When asked for an overall assessment of the operation 25 patients thought it had been successful and would recommend it.

### DISCUSSION

Insertion of malleable penile rods is an established surgical option in the treatment of impotence (Gottesman *et al.* 1977, Moul and Mcleod 1980, Krane *et al.* 1981, Subrini 1982). Our initial assessment did not distinguish between organic and non-organic causes as this preceded the organisation of our sleep laboratory. The very first patient in this series was a non-diabetic recently divorced teacher without any obvious underlying disease. We believe this was the only case of non-organic impotence. The majority of our patients were diabetic. The younger mean age of the insulin dependent diabetics may explain the shorter duration of impotence before presenting themselves when compared to those of maturity onset. The subset of post-surgical impotence included two patients of neurogenic origin – one, a chronic alcoholic who developed impotence and paraparesis following an intradural abscess. The limb weakness improved after surgical drainage but the impotence persisted. The other patient had pelvic denervation following abdomino-perineal resection for carcinoma of rectum. Two patients had hypogonadism following hypophysectomy for acromegaly and bilateral orchidectomy for carcinoma of prostate though the former had a normal hormone profile. In three patients there were severe medical conditions of aetiological importance. One was a hypertensive who had been difficult to control on various anti-hypertensives including ganglion blockers and another had severe angina. Both were probably of vasculogenic origin with drug contribution in the former. A third patient had chronic respiratory disease which is known to be associated with impotence in the absence of the usual known causes though the mechanism is less distinct (Fletcher and Martin 1982). Two patients had only Peyronie's plaques with painful, distorted erections with distal flaccidity which made insertion of rods a more appropriate treatment than Nesbit's procedure (Raz *et al.* 1977, Subrini 1984). One patient on dialysis developed a priapism which proceeded to cavernosal fibrosis having failed to respond to corporo-glandular and saphenocavernous shunt decompression. Our initial coronal approach followed the description of the procedure by Udo Jonas (1980). The limitations of this approach especially in cavernosal fibrosis requiring additional dorsal incision has been emphasized (Melman 1976). Its main advantage is access to the distal end of cavernosal bodies ensuring good support for the glans during intercourse. The peno-scrotal approach (Barry and Seifert 1979) which is now our standard approach gives adequate access to the distal and proximal parts of the corpora carvenosa enabling relatively easy dilatation and accurate sizing before selection of the appropriate set of rods (figure 2). The single instance of severe infection emphasizes the need for meticulous preparation, adequate antibiotic prophylaxis and aggressive treatment of early signs of infection in the post-operative period. Removal of the prostheses on account of partner disapproval was a salutory example emphasizing the importance of preoperative counselling and approval of both partners (Schoenberg *et al.* 1982, Beaser *et al.* 1982). The result of the survey demonstrates the general acceptance of the semi-rigid implant despite the initial post-operative discomfort observed also in other studies (Gotteman *et al.* 1977, Smith *et al.* 1979). Warning patients beforehand may improve tolerance of the pain and perhaps shorten the duration.

### CONCLUSION

This modest series from Southmead Hospital indicates once again the effectiveness and low complication rate of insertion of semi-rigid penile rods in the surgical treatment of impotence where non-operative treatment has failed or is inappropriate.

### REFERENCES

1. GOTTESMAN, J. E., KOSTERS, S., SAKTI, D., KAUFMAN, J. J. (1977) The Small–Carrion Prosthesis for Male Impotency. *J. Urol.* **117**, 289–290.
2. MOUL, J. M., MCLEOD, D. G. (1980) Experience with the AMS 600 Malleable Penile Prosthesis. *J. Urol.*, **135**, 929–931.
3. KRANE, J. R., FREEDBERG, P. S., SIROKY, M. B. (1981) Jonas Silicone-Silver Penile Prosthesis: Initial Experience in America. *J. Urol.* **126**, 475–476.
4. SUBRINI, L. (1982) Subrini Penile Implants. *Eur. Urol.* **8**, 222–226.
5. FLETCHER, E. C., MARTIN, R. J. (1982) Sexual Dysfunction and Erectile Impotence in Chronic Obstructive Pulmonary Disease. *Chest* **81**, 413–421.
6. JONAS, U., JACOBI, G. H. (1980) Silicone-Silver Penile Prosthesis: Description, Operative approach and results. *J. Urol.* **123**, 865–867.
7. BARRY, J. M., SEIFERT, A. (1979) Peno-scrotal approach for placement of paired penile implants for impotence. *J. Urol.* **122**, 325.
8. MELMAN, A. (1976) Experience with implantation of the Small-Carrion penile impotence for organic impotence. *J. Urol.* **116**, 49.
9. SCHOENBERG, H. W., ZARNS, C. K., SEGRAVES, R. T. (1982) Analysis of 122 unselected impotent men subjected to Multidisciplinary evaluation. *J. Urol.* **127**, 445–447.
10. BEASER, R. S., VAN DER HOEK, C., JACOBSON, A. M., FLOOD, T. M., DESAUTELS, R. E. (1982) Experience with penile prosthesis in the treastment of impotence in Diabetic Men. *J.A.M.A.* **248**, 943–948.
11. RAZ, S., DEKERNION, J. B., KAUFMAN, J. J. (1977) Surgical treatment of Peyronie's disease: A new approach. *J. Urol.* **117**, 598–601.
12. SUBRINI, L. (1984) Surgical treatment of Peyronie's disease using penile implants: Survey of 69 patients. *J. Urol.* **132**, 47–50.

**Figure 1**

Age distribution.

**Figure 2**

Aetiologies.

**Figure 3**

Peno-scrotal approach for insertion of rods.

**Figure 4**

Jonas-Koss semi-rigid rods.

## Figures and Tables

**Figure 1 f1:**
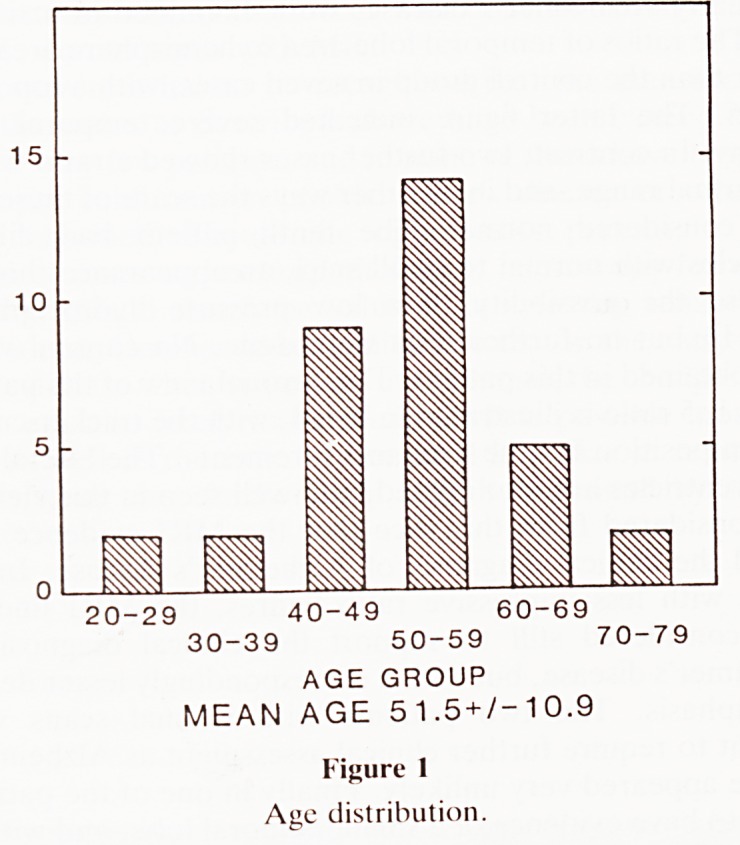


**Figure 2 f2:**
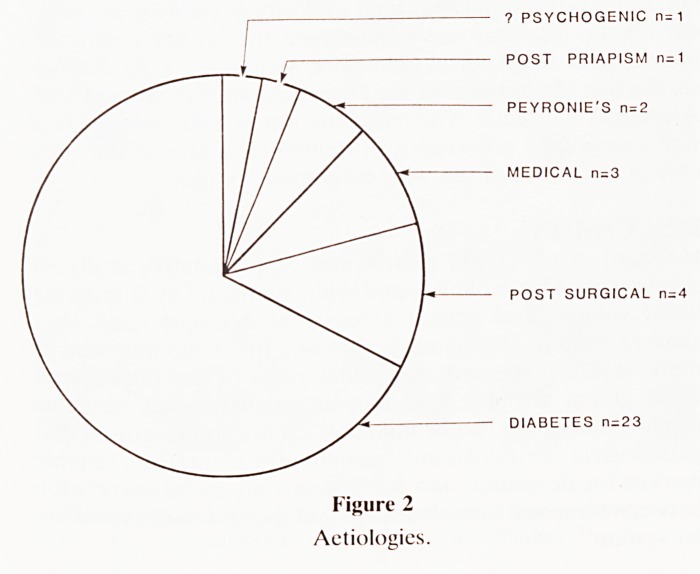


**Figure 3 f3:**
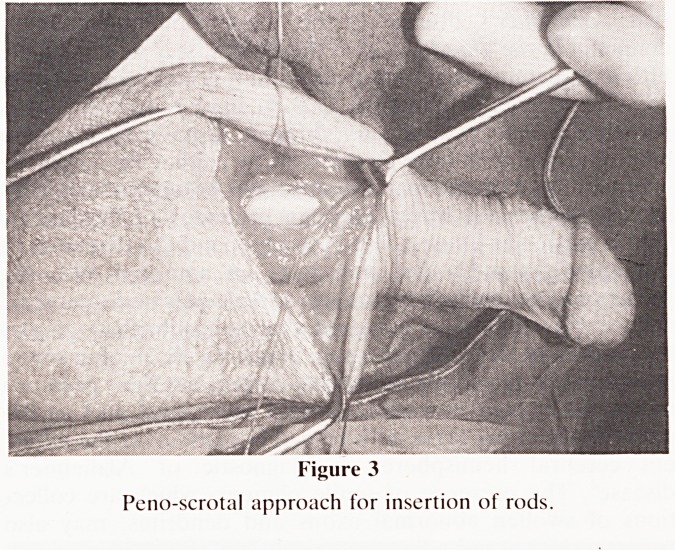


**Figure 4 f4:**